# A Possible Role of Prolonged Whirling Episodes on Structural Plasticity of the Cortical Networks and Altered Vertigo Perception: The Cortex of Sufi Whirling Dervishes

**DOI:** 10.3389/fnhum.2017.00003

**Published:** 2017-01-23

**Authors:** Yusuf O. Cakmak, Gazanfer Ekinci, Armin Heinecke, Safiye Çavdar

**Affiliations:** ^1^Department of Anatomy, School of Biomedical Sciences, University of OtagoDunedin, New Zealand; ^2^Radiology Department, School of Medicine, Marmara UniversityIstanbul, Turkey; ^3^Brain Innovation BV, Biopartner CenterMaastricht, Netherlands; ^4^Department of Anatomy, School of Medicine, Koc UniversityIstanbul, Turkey

**Keywords:** cortex, whirling, meditation, vertigo, vestibular, plasticity

## Abstract

Although minutes of a spinning episode may induce vertigo in the healthy human, as a result of a possible perceptional plasticity, Sufi Whirling Dervishes (SWDs) can spin continuously for an hour without a vertigo perception.This unique long term vestibular system stimulation presents a potential human model to clarify the cortical networks underlying the resistance against vertigo. This study, therefore, aimed to investigate the potential structural cortical plasticity in SWDs. Magnetic resonance imaging (MRI) of 10 SWDs and 10 controls were obtained, using a 3T scanner. Cortical thickness in the whole cortex was calculated. Results demonstrated significantly thinner cortical areas for SWD subjects compared with the control group in the hubs of the default mode network (DMN), as well as in the motion perception and discrimination areas including the right dorsolateral prefrontal cortex (DLPFC), the right lingual gyrus and the left visual area 5 (V5)/middle temporal (MT) and the left fusiform gyrus. In conclusion, this is the first report that warrants the potential relationship of the motion/body perception related cortical networks and the prolonged term of whirling ability without vertigo or dizziness.

## Introduction

Most studies to date that have investigated brain networks for vertigo or vestibular system have used or focused on the sole stimulation of the vestibular system, such as caloric or galvanic stimulation. However, these methodological approaches lack an engagement of proprioception and vestibular organ stimulation that would mimic motion perception based vertigo. To date, there is not a study that has investigated the structural plasticity induced by a prolonged period of stimulation using both systems, together. The Mevleviye Semazens, alternatively known as Sufi Whirling Dervishes (SWDs), have a unique meditation style that is termed as the Sema Ceremony which may provide a unique model to investigate cortical networks of motion perception and balance together with vestibular and proprioception sensory systems.

The Mevleviye is an ascetic Sufi order founded in 1273 in Konya, Turkey (Smeets, 2006). In the Sema Ceremony, a SWD rotates anti-clockwise around the vertical axis of their body, while also rotating around the other SWDs. Whirling intends to be a travel of soul awareness and loosening of the material self. To become trained for the Sema Ceremony so that they can able to rotate up to 1 h without vertigo or dizziness perception, Sufis traditionally receive up to 1000 days of training within the Mevlevi houses. At the end of that time, the Sufis are now trained as SWD. They re-join their families and return to their jobs, but gather together for Sema ceremony for several days in a week (Smeets, 2006). This unique whirling based meditation style of SWDs achieves extraordinary physiological outcomes that overcome vertigo and balance impairment, which would be expected after prolonged times of whirling.

It has been argued that alternating self-localization is caused by abnormal integration of vestibular signals. Additionally, it has been shown that vestibular processing is involved in space perception and locomotion (somatosensory processing) as well as the cognitive aspects of own-body representations, the consciousness of the own-body and bistable visual perception (Lopez et al., 2010, 2012).

The default mode of functioning was initially defined on particular areas of the brain that decrease activity when subjects focus on goal-directed tasks in comparison to simply resting (Raichle and Snyder, 2007). In the following years, the default mode definition extends to default mode network (DMN; Raichle and Snyder, 2007). The DMN of the brain has been observed to be related to self-awareness, consciousness, embodiment and also unhappiness (Killingsworth and Gilbert, 2010; Brewer et al., 2011). Therefore, it may also be theorized that prolonged periods of whirling based meditation of the Sufi dervishes contribute to structural changes in the networks of the DMN and self-perception, as well as motion perception related networks. A recent analysis of research on DMN that included anatomical connectivity and task-evolved neuroimaging revealed hubs of the resting state activity, including posterior cingulate cortex (PCC) and Precuneus areas (Gramann et al., 2006; Andrews-Hanna, 2012). It is relevant to note that in Kang’s study, the reported regions of decreased cortical thickness in meditators’ brains were the precuneus and PCC of the DMN (Kang et al., 2013). Additionally, a study that looked at functional magnetic resonance imaging (fMRI) scans of all meditation types but not including SWDs (Brewer et al., 2011) demonstrated that experienced meditators compared with controls showed decreased activity in the DMN including the main hub precuneus (Laird et al., 2009).

Previous studies demonstrated that long-term meditation practice is associated with altered resting brain activity which suggests long lasting activity changes persist in the brain (Lutz et al., 2004). The following cross-sectional studies demonstrated that meditation and experience dependent differences are correlated with cortical thickness (Maguire et al., 2000; Mechelli et al., 2004; Lazar et al., 2005). Significant positive associations were also evidenced between the cognitive ability factor and cortical thickness in most multimodal association areas in a large sample of healthy children and adults (Karama et al., 2009). Moreover, a relationship between cortical thickness and functional activation in the early blind have also been demonstrated recently (Anurova et al., 2014). A recent research (Burge et al., 2016) demonstrated that cortical thickness in human V1 is associated with central vision loss in 10 macular degeneration patients in comparison to 10 controls in a cross-sectional study that underlines the functional relationship with the cortical thickness. Considering the motionless but embodiment-related meditation study results that demonstrated a functional depression on cortical hubs of body perception networks, cortical thickness changes in experienced based cross-sectional studies and loss of visual input reflections on thinner relevant cortical areas, we theorized that there might also be a decrease in cortical thickness of precuneus and PCC of the DMN in SWDs as a results of the depressed or altered perceptions of motion and embodiment inputs to induce the cortical neuronal changes, resulting in the thinning of responsible cortices. Any additional structural plasticity findings of the SWDs’ cortical areas may also have the possibility to highlight the plasticity of the motion related networks that may be responsible for the alterations of the vertigo perception of SWDs. Improvement of navigation softwares for non-invasive brain stimulation techniques like Transcranial Magnetic Stimulation (TMS) have enabled structural and functional mapping of the brain and targetted stimulation of the specific cortical areas to be performed. This study aimed to map the structural cortical plasticity induced by Sufi whirling meditation as a unique human model of vestibular system stimulation and plasticity to clarify a network that may alter vertigo perception in SWDs and it may also provide a potential cortical map for non-invasive brain stimulation modalities to alleviate vertigo.

## Materials and Methods

### Participants

It is noted that far fewer SWD ceremonies are performed in Mevlevi-houses, as a result of secularization policies enforced by government in the early 20th Century. Although, in the late 20th Century, the Turkish government did again allow performances, most of these have been confined to public tourist audiences and are simplified to meet commercial requirements (Smeets, 2006). Consequently, it is difficult to select SWDs who perform the whirling ceremony using the traditional physical and spiritual method.

Ten (8 male, 2 female adults) right-handed traditional SWDs with greater than 8 years and an average of 10.5 years of whirling meditation (regular two whirling sessions each week) and 10 (8 male, 2 female adults) meditation naive right-handed controls were included into our study. The controls were case-matched for the country of origin and location (Turkey, Istanbul), primary language (Turkish) and demographics such as sex, age, race, education and employment status (SWD mean age: 32 years (range: 26–44), 8 male, 2 female. Control group mean age: 33 years (range: 26–44), 8 male, 2 female). Exclusion criteria for all subjects were abnormalities in magnetic resonance imaging (MRI), MRI incompatible implants and implanted devices and general medical disorders or any clinically relevant abnormalities. All subjects were free of medical, neurological and psychiatric disorders.

All procedures of this study were carried according to the principles and procedures outlined in the Declaration of Helsinki for Medical Research involving human subjects. The study was approved by the Ethics Committee (Prof. Ihsan Solaroglu - Head of the ethical committee for non-invasive human research. Institutional Review Board) at the Koç University. Each Participant provided written informed consent before entering the study and understood that s/he could discontinue the study at any time.

### Scanning Sequence

MRI scans of the participants were carried out on a 3T scanner with an 8-channel head coil (MAGNETOM Verio, Siemens Healthcare, Erlangen, Germany). The three-dimensional magnetization prepared rapid acquisition gradient echo sequence (3D T1-weighted MPRAGE). This was used to acquire the volume data of the whole brain of all the participants. 3D T1-weighted MP-RAGE protocol (42) was used with the following parameters: TR = 1670 ms, TE = 2.47 ms, TI = 900 ms, flip angle 9°, 176 slices scanned for sagittal plane with 1.0 mm slice thickness and the scanning matrix was 256 × 256 with a field of view of 250 mm, resulting in a voxel size of 1.0 mm × 1.0 mm × 1.0 mm. Total scan time for 3D T1-weighted imaging was 3.47 min.

### Anatomical Data Processing

#### Data Import, Preprocessing and Normalization

Raw MRI data from each subject was provided in DICOM format. It was then imported and converted into BrainVoyager’s internal “VMR” data format. Correction for inherent spatial intensity inhomogeneities was applied according to Vaughan et al. (2001). The data was then transformed into AC-PC position and Talairach (TAL) standard space.

#### Cortex Segmentation

Segmentation of the gray/white matter boundaries was achieved using the method of Kriegeskorte and Goebel, 2001. This included automatic segmentation routines, followed by a “bridge removal” algorithm, which ensured the creation of topologically correct mesh representations. For the two resulting segmented subvolumes, borders were tessellated to produce a surface reconstruction of the left hemisphere (LH) and right hemisphere (RH; Kriegeskorte and Goebel, 2001). All processing steps for segmentation as well as cortical thickness calculation have been thoroughly checked and evaluated by an expert. This is an important part of the preparation for the cortical thickness calculation in BrainVoyager, which is not directly comparable to automated analysis approaches in other analysis tools.

#### High-Resolution Intersubject Cortex Alignment

A high-resolution, multiscale cortex alignment procedure was performed following the method of van Atteveldt et al. (2004). This procedure substantially increased the statistical power and spatial specificity of group analyses. Before performing the group analysis on the basis of the subject-specific cortical thickness maps, all the single subject maps have been aligned using transformation matrices generated on the basis of cortical alignment (Fischl et al., 1999). The Cortex-based alignment approach (Fischl et al., 1999) has been specifically applied to the data to allow a proper comparability between cortical structures between subjects. Taken this into account, smoothing the cortical thickness data was not necessary. The addition of smoothing may even prove to be more harmful than helpful. In this context, multiple reference articles using BrainVoyager for analyzing cortical thickness data don’t apply spatial smoothing (Davis et al., 2008; Geuze et al., 2008; Strenziok et al., 2011; Van Swam et al., 2012; Thorns et al., 2013).

#### Cortical Thickness Analysis

The normalized version of each VMR was prepared in the following way to prepare the calculation of cortical thickness. First, the VMR data was interpolated to a higher resolution (0.5 mm * 0.5 mm * 0.5 mm) version using a sinc interpolation. In this new dataset, the ventricles and subcortical areas were filled, using a standard intensity value. Using an automatic detection approach, the cerebellum was removed. By applying a sigma filtering step, the tissue contrast of the data was enhanced.

Next, the boundary between gray and white matter was detected using a gradient-based adaptive approach. On the basis of a dilation procedure, the border between gray matter and Cerebrospinal fluid was detected. The final result of the preparatory steps consists of a VMR representing only gray and white matter in two grayscale/intensity values. To improve the quality of the procedure, this dataset was compared to the original VMR file in 0.5 mm resolution and corrected for potential errors.

To calculate the cortical thickness in the whole cortex, Laplace equations (Jones et al., 2000) were applied. The volumetric cortical thickness values were sampled to standardized surface meshes of the separate hemispheres using trilinear interpolation.

To correct the final result map showing group differences in cortical thickness, the automated cluster-level thresholding approach (Forman et al., 1995) was applied. This means that every patch of interest (POI) exceeding a calculated size (square mm on the surface mesh) is considered significant. The final result map has on average a corrected false alarm level of 5%. A cluster defining the threshold (CDT) of *p* < 0.05 was utilized. After applying the cluster level thresholding method, each contiguous regions of interest on the surface reaching a size beyond the calculated region size threshold (58 square mm for the left and 65 square mm for the RH) was turned into a POI. Based on the idea that there was no specific assumption about the direction of a difference in cortical thickness between groups, a two-sided *t*-test was performed to analyze the group differences.

For the surface-based analysis, a standardized mesh size was applied to the cortical surface of every subject. The standardized mesh has exactly “40961” vertices per hemisphere (identical for every subject) and thus solves a potential mapping issue between regions and between subjects. For the group comparison of the subject-specific cortical thickness surface maps, a simple subtraction of the cortically aligned thickness values was performed between two cohorts (Sufi dervishes and a control cohort). The analysis was performed specifically for each of the hemispheres based on the standard approach of segmentation and cortex-based alignment in BrainVoyager QX. The separation of hemispheres also allows for a more specific evaluation and analysis of hemisphere-specific effects. The variability within and between groups was also checked besides just using a *t*-test to compare the groups. It is still important to check the details within globally “detected” regions of interest. We have done this to perform a “sanity check” of the data included within the analysis and to explore the variability within and between groups.

## Results

**Figure 1:**
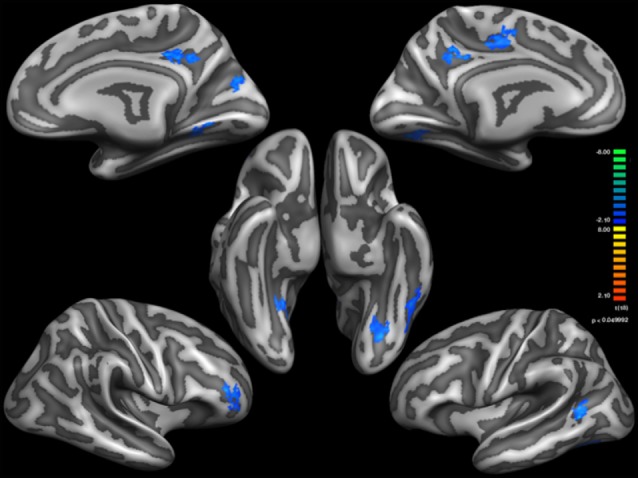
**Thinner Cortical areas in Sufi Dervishes (Sufi > Controls).** All regions are based on cluster thresholding.

Figure 1 shows regional differences in cortical thickness between Sufi dervishes and control group displayed on the average surface mesh (after applying the cortex-based alignment procedure to obtain optimal fitting of cortical structures). An average difference in cortical thickness of 0.10 mm for the LH and 0.15 mm for the RH was found. We compared the average cortical thickness within each hemisphere between the groups and found no significant differences within either of the hemispheres (LH: *t* = 1.56, *p* = 0.14, RH: *t* = 2.07, *p* = 0.055).

Table 1 shows the cortical thickness differences between Sufis and Controls for the whole brain and Table 2 shows the significant clusters with their *t, p* values, *X,Y,Z* coordinates and areas on both hemispheres.

**Table 1:** **The cortical thickness differences between Sufis and Controls for the whole brain**.

	Sufis	Controls	Difference
**LH**
Average thickness (mm)	2.92	3.02	−0.10
Maximum thickness (mm)	3.11	3.28	−0.17
Standard deviation	0.16	0.15	0.01
**RH**
Average thickness (mm)	2.90	3.05	−0.15
Maximum thickness (mm)	3.20	3.22	−0.02
Standard deviation	0.19	0.13	0.06

*LH, Left hemisphere; RH, Right hemisphere*.

**Table 2:** **The significant clusters with their *t*, *p* values, *X*, *Y*, *Z* coordinates and areas on both hemispheres**.

	Cortical area	TAL *X*	TAL *Y*	TAL *Z*	*t*	*p*	Square mm
**Left hemisphere**
POI 1	Fusiform gyrus, temporal lobe	−42	−48	−10	−3.9369	0.0010	76.90
POI 2	Fusiform gyrus, occipital lobe	−27	−66	−5	−3.9400	0.0010	111.90
POI 3	MT/V5	−48	−50	5	−4.6672	0.0002	61.15
POI 4	PCC	−12	−19	43	−4.0061	0.0008	92.80
POI 5	Precuneus, PCC	−8	−35	33	−3.8878	0.0011	65.33
**Right hemisphere**
POI 1	Lingual gyrus	14	−53	−2	−4.2423	0.0005	80.88
POI 2	precuneus, parieto-occipital sulcus	11	−66	19	−3.8815	0.0011	74.91
POI 3	PCC, precuneus	5	−33	36	−4.6599	0.0002	88.32
POI 4	DLPFC	39	35	20	−3.9253	0.0010	76.8

On the basis of the detected POIs on the surface mesh, corresponding TAL coordinates were extracted. This is based on the referential connection between the underlying TAL VMR and the cortical surface meshes created afterwards.

Based on external analysis of the coordinates using the “Talairach daemon” database (Lancaster et al., 1997) was performed. The results are displayed in Table 2. Figure 1 demonstrates surface maps for the statistically significant differences of cortical thickness between groups (Sufi–Controls) in both hemispheres : four POIs for the RH and five POIs for the LH.

## Discussion

The present study demonstrates differences in cortical thickness analysis between the 10 SWDs brain and 10 control cases as a proof of structural plasticity potentially induced by whirling meditation of SWDs.

Cortical thickness analysis, as structural plasticity reflection, is considered one of the best tools to reveal prolonged effects of meditation on the cerebral cortex. It has been well documented in the literature that meditation exercises including Zen meditation induce cortical plasticity in specific cortical zones as thickened gray matter (Lazar et al., 2005; Pagnoni and Cekic, 2007; Hölzel et al., 2008, 2010; Luders et al., 2009; Vestergaard-Poulsen et al., 2009; Grant et al., 2010, 2013). While the study by Lazar et al. (2005) demonstrated increased cortical thickness in the insula and prefrontal regions, yet Kang et al. (2013) demonstrated increased cortical thickness in the frontal and temporal regions only, and decreased cortical thickness in the DMN including the main hubs precuneus and PCC with different type of meditations. These findings are supported by fMRI studies by Brewer et al. (2011). Brewer et al. (2011) demonstrated decreased activity in the hubs of the DMN after numerous types of meditation. Cortical thickness results of the SWDs in this study showed similar results to Brewer et al. (2011) and Kang et al. (2013) reports for the DMN plasticity. There were four thinner cortical areas in the RH, and five thinner in the LH including the hubs of DMN as precuneus and PCC. There were no thicker cortical areas compared with the control group. The thinner cortical zones of SWDs were the Precuneus, PCC and on both hemispheres and middle temporal (MT)/visual area 5 (V5) and fusiform gyrus on the left and Dorsolateral Prefrontal cortex (DLPFC) and lingual gyrus on the RH. With the SWDs having meditation practice combined with movement, the results obtained in the left MT/V5, fusiform gyrus and Right DLPFC and DMN hubs may all underline the altered vertigo perception in whirling motion of SWDs.

### Altered Perception of Motion and Vertigo

#### Left MT/V5

Our results demonstrated a thinner Left MT/V5 in the LH of the SWDs. In previous studies that have explored V5/MT, the left MT/V5 was shown to have more robust involvement in motion detection (Beckers and Hömberg, 1992; Stewart et al., 1999; Antal et al., 2004; Schwarzkopf et al., 2011; Tadin et al., 2011; Murd et al., 2012).

The dominance of left V5/MT may explain the thinner V5/MT on the LH of the SWDs. With the depressed function of left V5/MT, the perception of the movement during whirling meditation may be depressed and as a consequence, the possible physiological and motor responses to whirling motion perception may be inhibited. In addition to robust function of left V5/MT on motion perception, discrimination function is also found to be related with the left V5/MT rather than the right V5/MT (Cornette et al., 1998).

#### Fusiform Gyrus, Right DLPFC and Change Detection

Fusiform gyrus is well known for its face detection function but it also discriminates between places; particularly in the medial and anterior portions of the fusiform gyrus. Conscious detection of visual changes including face and place changes mostly relies on regions of the ventral visual cortex including the fusiform gyrus, but also the right DLPFC (Beck et al., 2001). The left fusiform gyrus is also found to be related with the non-face related visual changes (Rangarajan et al., 2014). Further, left fusiform gyrus showed greater activity when participants attended to changes in face parts than to changes in whole face. The opposite pattern was demonstrated in the right FFA (Rossion et al., 2000).

The role of right DLPFC in visual change awareness has been demonstrated by fMRI and TMS studies (Beck et al., 2001; Turatto et al., 2004). The right DLPFC is activated with place changes and it has been shown to be non-active in the cases of change blindness by fMRI and TMS studies (Beck et al., 2001). Additionally, it is demonstrated that activation of ventral occipitotemporal cortex, including lingual gyrus, is also related to the processing of visual information for human faces (McCarthy et al., 1991).

Our analyses showed that in the SWDs cortex, the fusiform gyrus were thinner on the left and lingual gyrus and DLPFC were thinner only on the right side. By doing so, SWDs may also have an altered state of place change perception, especially important for keeping the body stability in the case of a whirling meditation which includes a continuous place change stimulation for the place change detection areas of fusiform gyrus, lingual gyrus and right DLPFC.

#### Precuneus, Egocentric Framework and Default Network Hubs

In three dimensional spaces, humans navigate themselves with the aid of spatial relationship references. Two different type of spatial reference coding frameworks (or frame of reference) are *allocentric* and *egocentric* abilities. While the *allocentric* ability depends on object to object positional references and is independent from self-position, the *egocentric* ability depends on self to object positional references (Vogeley and Fink, 2003; Gramann et al., 2006). Research on the cortical regions related to these two distinct frameworks has revealed that egocentric conditions have activations exclusively within the precuneus in comparison to allocentric conditions (Gramann et al., 2006). The precuneus as a location of the egocentric representation does the updating during self-motion and it is demonstrated that it is the only region for working memory of directional updating (Land, 2014). The precuneus is considered as a machinery of self-perception that builds a conscious self-perception by providing continous data of external space to maintain a synchronous relationship with the body in the move and the objects in the environment (Land, 2014). In this study, bilateral thinner precuneus in SWDs was shown. Thinner precuneus may underline the role of egocentric framework depression to aid the extraordinary whirling ability of SWDs in addition to the role of depressed activations of left MT/V5 and fusiform gyrus and right DLPC and lingual gyrus. The last but not the least, it has been shown that the electrical cortical stimulation of the precuneus produce vestibular sensations and implicating the role of precuneus for vestibular information processing (Wiest et al., 2004).

#### The Default Mode and Subsystems

A recent analysis of the DMN neuroimaging studies suggests the precuneus is the core node or the hub of the DMN (Andrews-Hanna, 2012). Activation of both the DMN core hub precuneus and prefrontal cortex has been found to be related to self-perception and mind wandering (Kjaer and Lou, 2000; Kjaer et al., 2002). Imaging studies have shown that these areas are deactivated in the case of altered states of consciousness including vegetative state, hypnosis and sleeping (Maquet et al., 1997, 1999; Laureys et al., 1999; Hobson et al., 2000; Maquet, 2000). It has also been observed that the precuneus is one of the first zones to be reactivated in the case of a reconsciousness (Laureys et al., 2004, 2006).

In addition to bilateral thinner precuneus in SWDs, the posterior cingulate gyrus was thinner. In the context of thinner precuneus and posterior cingulate gyrus in SWDs’s brains, it has been shown that these becomes progressively deactivated in anesthetic sedation states (Alkire et al., 1999; Fiset et al., 1999). Additionally, independent researchers have observed that coactivation of precuneus and PCC occurs when processing intentions related to self (Vogeley and Fink, 2003; den Ouden et al., 2005).

The outcomes of the present study may open a new era in the field of vertigo therapy. This is because if whirling based movement achieves inhibition of the self-perception and motion related regions such as left V5/MT, fusiform gyrus, right DLPFC precuneus and PCC and the DMN hub, then this may underline the cortical plasticity for a network of resistance to vertigo perception. Targeting these areas to inhibit by interventional non-invasive brain stimulation tools may achieve maintenance of balance when conditions of extreme spatial and proprioception information occur in addition to pathological conditions that trigger vertigo.

### Mood

A possible mood enhancing effect of the defined structurally plastinated cortical areas in the present study is worth to give an attention.

In the theory of mind, the DMN activity as a self-awareness state is correlated with the neuronal representation of mind-wandering (Kjaer and Lou, 2000; Kjaer et al., 2002). This DMN is active at all times except when suppressed by other networks, stimulated by other states, and its activity is correlated with lower levels of Happiness (Killingsworth and Gilbert, 2010; Brewer et al., 2011). Gusnard and Raichle (2001) demonstrated that the goal-directed cognitive process can decrease the activity of precuneus, the core hub of the DMN or mind-wandering network of the brain. Therefore, it is theorized that prolonged periods of goal-directed cognitive processes may decrease the mind-wandering activity in the SWD’s brain because the precuneus activity has been decreased. fMRI data results from the Brewer et al. (2011) study on the DMN of the experienced meditators showed that the DMN main nodes, including medial prefrontal cortex and PCC areas extending to precuneus were relatively deactivated. Further, precuneus and PCC have been observed as thinner and concluded to be so as a response to meditation (Kang et al., 2013). In line with these results, the structural cortical thickness analysis of the two cohorts in this study showed that the experienced SWDs had bilateral thinner PCC and precuneus zones. It can be therefore be theorized that the prolonged period of decreased activity in the PCC and precuneus may result in the thinner zones in the SWDs. As the DMN activity presented thinner in the SWDs, this is likely related to suppressed mind wandering, and as a consequence, this plasticity may improve the happiness level in SWDs. These results justify further studies to clarify the potential effects of SWDs’s unique meditation on their moods and depression levels.

### Behavior

In addition to potential mood enhancing effects by Whirling Meditation as achieved with decreased mind wandering, or by DMN activity as achieved with other types of meditation, decreased activity in the DLPFC may contribute to the behavioral attribute of honesty. As the precuneus stands as a core hub for the DMN, the DLPFC stands as a core hub for the executive network (Beaty et al., 2015). It has been shown that to achieve creative idea production, there is a coupling of the PCC and precuneus of the DMN with the right DLPFC of the executive network (Beaty et al., 2015). It has also been shown that increased task complexity or increased rule complexity is accompanied by increased activation in the right DLPFC and precuneus (Jia et al., 2015). In the case of lying, we need a creative idea production process and to consider long-term benefits. It has been shown that disruption of the right DLPFC leads to a greater selection of both gains and losses that have better immediate but worse long-term alternatives (Essex et al., 2012). It has also been shown that when the right DLPFC activity was disrupted using TMS, subjects were statistically less inclined to lie about the subject matter tested (Karton and Bachmann, 2011). Regarding the decreased thickness in the right DLPFC in SWDs, it may be theorized that this contributes to improve their behavioral attitude of honesty. It may also be speculated that the decrease in the thickness of fusiform and right DLPFC contributes to decreased discrimination of places and faces, and that such an altered perception of the world and people is also a result of SWDs’s meditations. Further studies are needed to investigate whether the suppression of cortical areas related with discriminational perception leads to less selfish, egocentric behavior and increased level of happiness.

### Neuroprotection

The DMN activity is also found to be related to Alzheimer’s disease (Bero et al., 2011). Bero et al. (2011) demonstrated that increased amyloid-β deposition overlaps with the DMN, including the core hub, precuneus and PCC regions. This overlapping is attributed to the higher metabolic activity of the DMN. The decreased thicknesses of these regions in SWDs cortex that are shown in the present study may underline a possible whirling meditation protective effect of over Alzheimer disease by its possible effect of decreasing the amyloid-β deposition in these regions because of their decreased activity in SWDs.

### Limitations

The current study used a cross-sectional design and it is performed on a small group of SWDs. As a result of the cross-sectional character of the study, the results are correlational and an absolute relationship between the cortical thinning and whirling experience can not be suggested. In addition, it may also be argued that individuals who have such cortical properties are more likely becoming a Sufi whirling Dervish. On the other hand, it is worth to note that there are numerous factors to consider that relate the outcomes of the present study to whirling experience. This is a cross-sectional study in a very unique (and rare) group who had traditional training for whirling (approximately 1 year in most of the cases) and each of them reported that they were falling down when they try to whirl in the first months of the whirling training sessions. This indicates that they did not have a unique previous ability that was superior to the predisposition of the control group. After the long-term training, they gained an ability to whirl without vertigo. The present cross-sectional study focused on experienced Sufis that passed through the same traditional whirling training that enabled them to whirl for an hour without falling. In this context, the detected structural differences are more likely to be specific to motion perception and body perception networks. As the analyses were performed without visual input, the outcomes of these areas are free of bias. Analyses within the article were almost not separated but discussed in the context of relevant networks. The structural cortical plasticity that was demonstrated in SWDs were distributed over the body/motion perception areas, therefore the discussion was focused on the possible relationships of the structural plasticity of the body/motion perception areas and their potential role to alter vertigo perception. In sum, we only explained the results on the basis of the previous work which was the most fitting way for the data obtained in this cross-sectional article. Longitudinal studies are needed to clarify the role of these areas in vertigo perception.

The outcomes of this cross-sectional study in a rare and unique group (whirling Sufi dervishes) warrants cortical zones which may have significant roles to alter vertigo/dizziness and address those areas for future studies. In conclusion, this is the first report that demonstrates correlations of the structural cortical plasticity and the prolonged period of vestibular system stimulation in humans.

## Author Contributions

YOC designed, performed the study, analyzed the data and wrote the manuscript and made critical review of the manuscript. GE performed the study, collected data, contributed analytical tool, made critical review of the manuscript. AH analyzed and interpreted data, contributed analytical tool, performed statistics and made critical review of the manuscript. SÇ contributed analytical tool, collected data, wrote manuscript and made critical review of the manuscript.

## Funding

The study is funded by the Start-up fund of YOC, School of Biomedical Sciences,University of Otago.

## Conflict of Interest Statement

The authors declare that the research was conducted in the absence of any commercial or financial relationships that could be construed as a potential conflict of interest. AH is employed by Brain Innovation at Biopartners Center, Maastricht, Netherlands, as a member of the Brain Voyager software support team. He assisted in preparing the data with the new version of BrainVoyager. AH’s employment by Brain Innovation does not alter the authors’ adherence to all the Frontiers Journals policies on sharing data and materials.

## Abbreviations

DLPFC: Dorsolateral Prefrontal Cortex
DMN: Default Mode Network
fMRI: Functional Magnetic Resonance Imaging
LH: Left hemisphere
MT: Middle Temporal
PCC: posterior cingulate cortex
POI: Patch of Interest
RH: Right hemisphere
SWDs: Sufi Whirling Dervishes
TAL: Talairach
TMS: Transcranial Magnetic Stimulation
V5: Visual area 5.
